# Morphological and morphometric specializations of the lung of the Andean goose, *Chloephaga melanoptera*: A lifelong high-altitude resident

**DOI:** 10.1371/journal.pone.0174395

**Published:** 2017-03-24

**Authors:** John N. Maina, Kevin G. McCracken, Beverly Chua, Julia M. York, William K. Milsom

**Affiliations:** 1 Department of Zoology, University of Johannesburg, Johannesburg, South Africa; 2 Department of Biology and Marine Biology and Ecology, Rosenstiel School of Marine and Atmospheric Sciences, University of Miami, Coral Gables, Florida, United States of America; 3 Department of Zoology, University of British Columbia, Vancouver, Canada; Vanderbilt University Medical Center, UNITED STATES

## Abstract

High altitude flight in rarefied, extremely cold and hypoxic air is a very challenging activity. Only a few species of birds can achieve it. Hitherto, the structure of the lungs of such birds has not been studied. This is because of the rarity of such species and the challenges of preparing well-fixed lung tissue. Here, it was posited that in addition to the now proven physiological adaptations, high altitude flying birds will also have acquired pulmonary structural adaptations that enable them to obtain the large amounts of oxygen (O_2_) needed for flight at high elevation, an environment where O_2_ levels are very low. The Andean goose (*Chloephaga melanoptera*) normally resides at altitudes above 3000 meters and flies to elevations as high as 6000 meters where O_2_ becomes limiting. In this study, its lung was morphologically- and morphometrically investigated. It was found that structurally the lungs are exceptionally specialized for gas exchange. Atypically, the infundibulae are well-vascularized. The mass-specific volume of the lung (42.8 cm^3^.kg^-1^), the mass-specific respiratory surface area of the blood-gas (tissue) barrier (96.5 cm^2^.g^-1^) and the mass-specific volume of the pulmonary capillary blood (7.44 cm^3^.kg^-1^) were some of the highest values so far reported in birds. The pulmonary structural specializations have generated a mass-specific total (overall) pulmonary morphometric diffusing capacity of the lung for oxygen (DLo_2_) of 0.119 mlO_2_.sec^-1^.mbar^-1^.kg^-1^, a value that is among some of the highest ones in birds that have been studied. The adaptations of the lung of the Andean goose possibly produce the high O_2_ conductance needed to live and fly at high altitude.

## Introduction

Biologists have long strived to understand how differences in the functional designs of the vertebrate respiratory systems correlate with metabolism, lifestyle and environment inhabited [1–4]. The adaptations that support avian flight at extreme altitude have been particularly perplexing [5–12]. Energetically, powered (active) flight is the most costly mode of locomotion [13–16]. It exacts various physiological and morphological adaptations [17]. Birds that fly at extreme altitude, the so-called ‘super birds’ [18], operate under very low ambient temperature and dry, rarefied and hypoxic air [19, 20]. To avoid the challenges presented by flight at high elevation, lowland resident birds that cannot tolerate extreme hypoxia [5] cover long distances to avoid flying over high obstacles [21].

The respiratory physiology of birds that fly at high altitude differs profoundly from that of low level flying ones [6]. For example, in the bar-headed goose (*Anser indicus*) [11, 12, 19], the Andean goose (*Chloephaga melanoptera*) [22, 23], the Tibetan chicken (*Gallus gallus*) [24] and the Ruppell’s griffon vulture (*Gyps rueppellii*) [25], mutations of hemoglobin have generated molecular configurations that increase O_2_ affinity [23–28]. Bar-headed geese in particular have evolved increased capacity of transporting O_2_ along the path from the atmosphere to the mitochondria [19]. Recently, it was shown that Andean geese, lifelong residents of the Andes, have evolved a different strategy of matching O_2_ supply to demand in hypoxic environments: while bar-headed geese match O_2_ supply and demand during rest in low O_2_ environments with large increases in ventilation [6, 28], the Andean geese exhibit very modest ventilatory response and instead greatly increase the amount of O_2_ extracted from each breath [29]. The latter suggests that Andean geese possess pulmonary structural adaptations that increase the capacity for O_2_ acquisition.

While the unique functional design of the avian respiratory system [30–39] undoubtedly increases O_2_ uptake efficiency, details on the structure of the lungs of the elite high-altitude flying birds are totally lacking. This is because of rarity of such species and the challenges posed in obtaining adequately fixed lung tissues. Of the known 63 species and 15 orders of volant and non-volant bird species on which pulmonary morphometric data exist [33, 36, 37, 39], none of them is an extreme high-altitude flyer. Here, we examined the lungs of the Andean goose, a large bird that lives at altitude between 3,000 and 6,000 meters of the Andes [40] to determine possible morphological specializations that may support its lifestyle.

The abbreviations used in the text are defined in Table 1 and the morphometric values are given as mean ±SD.

**Table 1:** Definition of abbreviations.

AC	Air capillary
BC	Blood capillary
BGB	Blood-gas (tissue) barrier
Deo_2_	Morphometric diffusing capacity of the erythrocyte
DLo_2_	Total (overall) morphometric pulmonary diffusing capacity of the lung
Dmo_2_	Membrane diffusing capacity of the total barrier, i.e., the blood-gas (tissue) barrier and the plasma layer
LP	Lung parenchyma
PL	Parabronchial lumen
S_(t)_	Surface area of the blood-gas (tissue) barrier
S_(t)_.V_(LP)_^-1^	Surface area of the blood-gas (tissue) barrier (S_(t)_) per unit volume of the lung parenchyma (V_(LP)_), i.e., the surface density of the respiratory surface area
V_L_	Volume of the lung
V_(LP)_	Volume of the lung parenchyma
V_(PCB)_	Volume of the pulmonary capillary blood
V_(PCB)_.S_(t)_^-1^	Volume of the pulmonary capillary blood (V_(PCB)_) per unit surface area of the blood-gas (tissue) barrier
τ_hb_	Harmonic mean thickness of the total barrier, i.e., the distance between the respiratory surface and the surface of the erythrocyte cell membrane (the air-hemoglobin pathway)
τ_ht_	Harmonic mean thickness of the blood-gas (tissue) barrier

## Materials and methods

### Ethical clearance, location and method of capture of the birds

Lungs were obtained from three wild Andean geese (*Chloephaga melanoptera*) captured and raised in San Pedro de Casta, Perú at an altitude of 3180 meters. Animals were collected under permit # 36087–2012 from the Gestión Florestral y de Fauna Sylvestre, Ministerio de Agricultura, Republica del Perú. They were two years old when sampled. All procedures were conducted according to guidelines approved by the Animal Care Committee at the University of British Columbia (A16-0019) in accordance with the Canadian Council on Animal Care.

### Fixation of the lungs

The birds were weighed and then killed by intravenous injection of propofol into the tibiotarsal vein (>20 mg/kg). With the body in a supine position, the lungs and the air sacs were fixed by intratracheal instillation with 2.3% glutaraldehyde buffered with sodium cacodylate (osmolarity 350 mOsm and pH 7.4) at a pressure head of 3000 Pa (1 cm H_2_O = 1 mbar = 10^2^ Pa). The top of the funnel, which was constantly topped up, was held 30 cm above the sternum of the supine bird. When it stopped flowing by gravity, the body wall was repeatedly gently squeezed to expel the air from the lung and the air sacs to achieve better penetration of the fixative. When the fixative finally stopped flowing, the trachea was ligated and the fixative left in the respiratory system for ~4 hours. Thereafter, the lungs were removed from their costovertebral attachments and immersed in fixative.

### Determination of the lung volume (V_L_)

In the laboratory, the extrapulmonary primary bronchus was trimmed close to the hilum and the adhering fat and connective tissue elements removed before the V_L_ was determined by the weight displacement method of Scherle [41].

#### Sampling of the lung and microscopic and morphometric analyses of the lung

Details on lung sampling, tissue processing for microscopy and morphometric analyses are given in Maina [42]. They are succinctly outlined below.

#### Sampling and analysis of the main structural components of the lung at the light microscopic level of magnification

The left lung of one of the birds was cut into five slices along the costal sulci and the slices in turn cut into halves just dorsal to the primary bronchus. The ten half-slices were processed and embedded in paraffin wax with the cranial face directed anteriorly. Sections were cut at 8 μm thickness and the first technically adequate slice stained with hematoxylin and eosin. The volume densities of the lung parenchyma (LP), the lumina of the parabronchi (tertiary bronchi) and the secondary bronchi, the blood vessels larger than blood capillaries (BCs) and the primary bronchus were determined field-by-field by point-counting using a 100-point Zeiss integrating graticule at a final magnification of x100. The absolute volumes of the structural parameters were calculated from the V_L_, the reference space.

#### Sampling and analysis of the structural components of the LP at the transmission electron microscopic level of magnification

The right lungs of the three birds were cut into five slices along the costovertebral sulci and the slices then cut into halves just dorsal to the primary bronchus. From the cranial surfaces of each of the ten half slices, 1 mm thick slices were cut and laid out flat. A transparent acetate paper with a quadratic lattice grid on which squares were numbered was then dropped onto the surface of the slice to avoid bias. Six random numbers that fell within the range of those on the part of the grid that lay on the slice were generated from a free computer software (https://www.random.org/integers) and small pieces of lung tissue taken from the areas (squares) where the numbers were located. When a random number fell on a large blood vessel or an airway (components of the lung that were analyzed at light microscopic level), an extra random number was generated until six pieces of tissue were sampled from the LP. The pieces of tissue were cut to a size of ~1 mm^3^ and processed for transmission electron microscopy. From the group of blocks processed from the pieces of tissue from each half slice of the lung (normally more than six in number), one block was picked at random and semithin sections cut and stained with toluidine blue. The block face was trimmed to an appropriate shape and size by removing the non-parenchymatous structures. Ultrathin sections were cut, mounted on 200-square wire-mesh coated copper grids and stained with lead citrate and counter-stained with uranyl acetate. For determination of the volume densities and the surface areas of the components of the LP, eight electron micrographs were taken from a predetermined corner (top right) of the grid (to avoid bias) from each section, at a primary magnification of x4,400. The same number of electron micrographs (80) was taken from the same areas of the grid squares at a higher primary magnification of x13,000 for the determination of the harmonic mean thickness of the blood-gas (tissue) barrier (τ_ht_) and the harmonic mean thickness of the total barrier, i.e., the distance between the respiratory surface and the erythrocyte cell membrane (τ_hb_). The images were enlarged by a factor of x2.5 and a quadratic lattice grid superimposed on top. The volume densities of the components of the LP, namely the BCs, the air capillaries (ACs) and the structural tissue of the LP were determined by point-counting, the surface areas by intersection counting and the τ_hb_ and the τ_ht_ by intercept length measurement using a logarithmic scale [43, 44]. The absolute volumes of components of the LP were calculated as the product of their volume densities and the volume of the LP, the reference space.

#### Morphometric modeling of the lung of the Andean goose for conductance of oxygen

After determining the relevant morphometric parameters, the membrane diffusing capacity (DMo_2_), the morphometric diffusing capacity of the erythrocyte (Deo_2_) and the total morphometric pulmonary diffusing capacity of the lung for oxygen (DLo_2_) were determined by applying the appropriate O_2_ permeation coefficient (Kto_2_) and the O_2_ uptake coefficient of the whole blood (Θo_2_) [43, 44]. The revised model of Weibel et al. [45] was used to determine the DMo_2_ and the DLo_2_.

## Results

### Morphological findings

**Fig 1:**
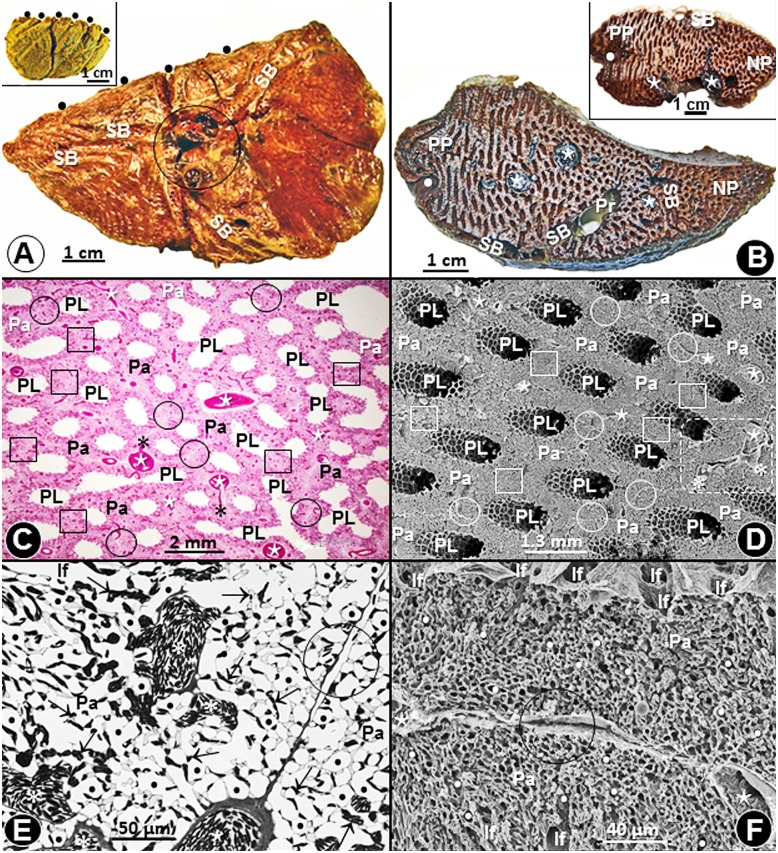
Lung, parabronchi and interparabronchial septa of the lung of the Andean goose (C*hloephaga melanoptera*). (A) Medial- and lateral (insert) views of the cone-shaped lung. •, costovertebral sulci; SB, secondary bronchi; circle, hilum. (B) Transverse slices of the lung showing large blood vessels (⋆), secondary bronchi (SB), a primary bronchus (Pr), paleopulmonic parabronchi (PP), neopulmonic parabronchi (NP) and a costovertebral sulcus (•). (C, D) Histological- (C) and scanning electron (D) micrographs showing parabronchial lumina (PL) that are surrounded by parenchyma (Pa). ⋆, interparabronchial blood vessels; *, intraparabronchial blood vessels; circle (O), areas where interparabronchial septa exist; square (□), areas where interparabronchial septa are missing. The area bounded by the dashed outline in D is enlarged on 2B. (E, F) Toluidine blue stained- (E) and scanning electron (F) micrographs showing interparabronchial septa (circle, O). Pa, parenchyma; ⋆, interparabronchial blood vessels; *, intraparabronchial blood vessels; •, air capillaries; ↑, blood capillaries; If, infundibulae.

**Fig 2:**
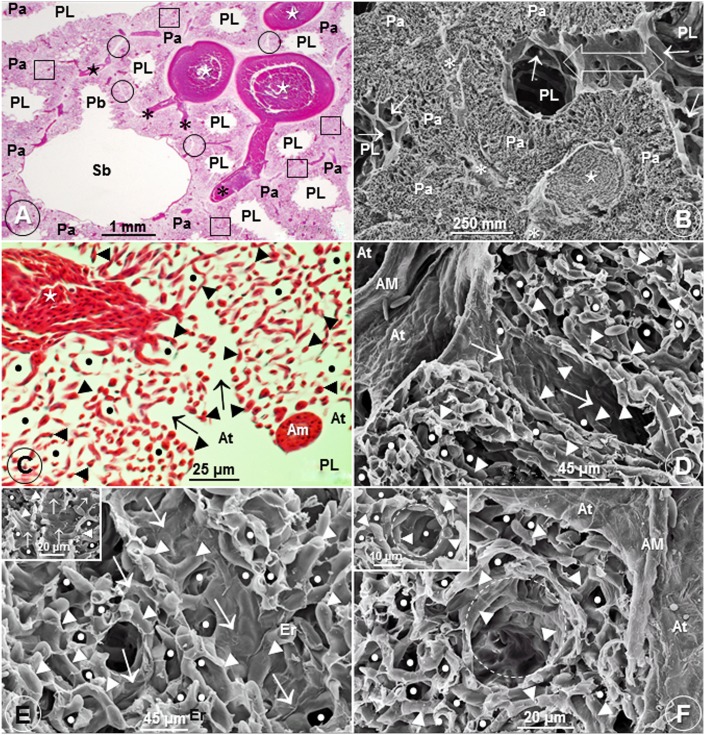
Parabronchi, interparabronchial septa and infundibulae of the lung of the Andean goose (C*hloephaga melanoptera*). (A, B) Histological- (A) and scanning electron (B) micrographs of the lung showing a secondary bronchus (Sb) giving rise to a parabroncus (Pb) (A) and an interparabronchial artery (⋆) giving rise to intraparabronchial arteries (*) (B). PL, parabronchial lumina; Pa, parenchyma; circle (O), areas where interparabronchial septa exist; square (□), areas where interparabronchial septa are missing; arrows, atria; open double sided arrow (B), area where adjacent parabronchi anastomose. The area shown in figure B is an enlargement of the dashed enclosed area in Fig 1D. (C-F) Histological- (C) and scanning electron (Figs D-F) micrographs showing the intense vascularization of the infundibulae (↑) (C-E) and dashed circles (F). PL (C), parabronchial lumen; ⋆ (C), intraparabronchial blood vessel; AM (D, F), atrial muscle; At (D, F), atria; ►, blood capillaries; •, air capillaries; Er (E), erythrocytes.

**Fig 3:**
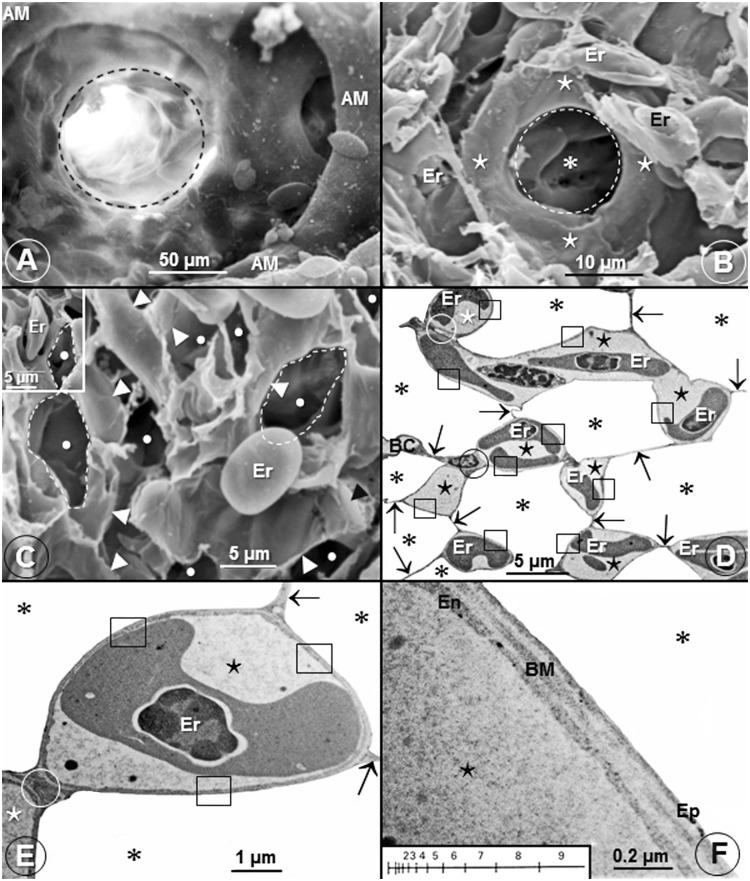
Infundibulae, air capillaries, blood capillaries and the blood-gas (tissue) barrier of the lung of the domestic fowl (*Gallus gallus* variant *domesticus*) and the Andean goose (C*hloephaga melanoptera*). (A) A scanning electron micrograph showing an avascular infundibulum (dashed circle) of the lung of the domestic fowl. AM, atrial muscle. (B) Scanning electron micrograph of the lung of the Andean goose (C*hloephaga melanoptera*) showing an air capillary (* encircled by a dashed circle) which is surrounded by a single blood capillary (⋆). Er, erythrocytes. (C) Scanning electron micrograph showing lung parenchyma with extravasated erythrocytes (Er) that correspond in size (diameter) with those of the air capillaries (•). Some of the air capillaries are outlined with dashes lines. •, air capillaries; ►, blood capillaries. In the insert, an erythrocyte (Er) has slotted into an air capillary. (D, E) Low- (D) and high (E) magnification transmission electron micrographs showing air capillaries (*) and blood capillaries (⋆). In E, a blood capillary (⋆) is almost completely surrounded by air in the air capillaries (*). ↑, epithelial-epithelial cells connections; □, blood-gas (tissue) barrier; Er, erythrocytes; circle (O), blood capillary-blood capillary connection. (F) High magnification transmission electron micrograph showing the structure of the blood-gas (tissue) barrier. It [blood-gas (tissue) barrier] consists of an epithelial cell (Ep), an endothelial cell (En) and a basement membrane (BM). ⋆, blood capillary; *, air capillary. The insert shows a logarithmic scale that was used to determine the harmonic mean thickness of the blood-gas (tissue) barrier and that of the total barrier.

The cone-shaped lungs (Fig 1A) of the Andean goose were firmly attached to the ribs and the vertebrae across six costovertebral sulci (Fig 1A and 1B). In some parts of the lung, interparabronchial septa were present while in others they were lacking (Fig 1C–1F). Interparabronchial blood vessels gave rise to relatively smaller intraparabronchial ones (Figs 1C–1E, 2A and 2B), the atria were conspicuous (Figs 1C, 1D and 2A–2D) and the infundibulae were intensely vascularized (Fig 2C–2F). This is an uncharacteristic feature of the lungs of birds that have so far been studied where the surface of the infundibulae is nonvascular (Fig 3A) with BCs being located in the LP. Probably to optimize respiratory surface area, occasionally, single BCs surround ACs (Fig 3B). Fig 3C shows an extravasated erythrocyte that measures ~13 μm in diameter lying next to an AC and one of the cells lodged into an AC (Insert). While most of the BCs connected by means of epithelial-epithelial cell retinaculae that also separated the ACs (Fig 3D and 3E), some BCs attached directly (Fig 3D and 3E). The blood-gas (tissue) barrier (BGB) comprised an epithelial cell, an endothelial cell and a common basement membrane (Fig 3F) and was rather uniform in thickness (Fig 3D–3F).

### Morphometric findings

The mean body mass and V_L_ of the Andean geese were respectively 2,636±144 g and 112±5.23 cm^3^ (S1 Table). The volume densities of the main structural components of the lung were: lung parenchyma (LP) = 61.7±7.4%; parabronchi and secondary bronchi = 25.8±3.7%; blood vessels larger than BCs = 9.5±1.4% and; primary bronchus = 3.0±5.2% (S2 Table). Concerning the structural components of the LP, the volume densities of the ACs, the BCs and the structural tissue were respectively 63.4±3.0, 28.2±2.9 and 8.4±0.13% (S3 Table). The surface area of the BGB was 2.52±0.025 m^2^ (S4 Table) and the τ_ht_ and the τ_hb_ (S5 Table), were respectively 0.222±0.01 μm and 0.474±0.08 μm and the Deo_2_, the DMo_2_ and the DLo_2_ (S6 Table) were respectively 0.221±0.02, 3.87±0.89 and 0.312±0.03 mlO_2_.sec^-1^.mbar^-1^.

**Fig 4:**
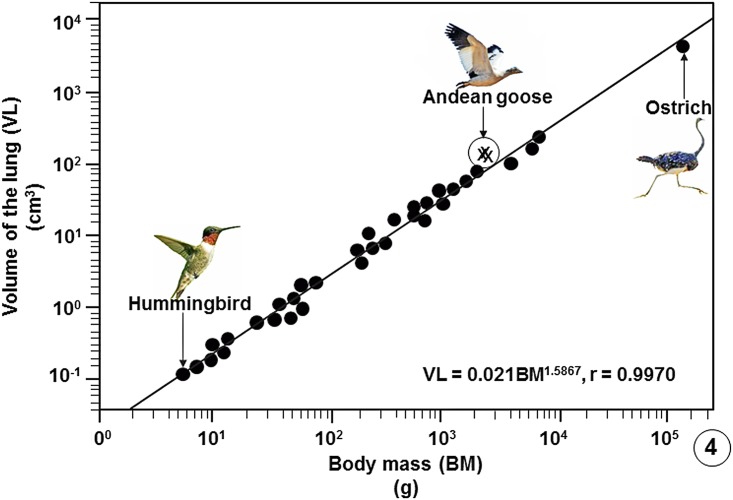
Regression line plotted on logarithmic x-y axes showing the correlation between the Volumes of the Lungs (VL) of birds on which data are available against Body Masses (BM). The values of the three specimens of the Andean goose (*Chloephaga melanoptera*) that were investigated in this study lie above the common regression line of the bird population. The data on which the regression line was plotted are summarized in publications [33], [37], [39] and [54–56] and are given in data supporting this paper (S8 Table).

**Fig 5:**
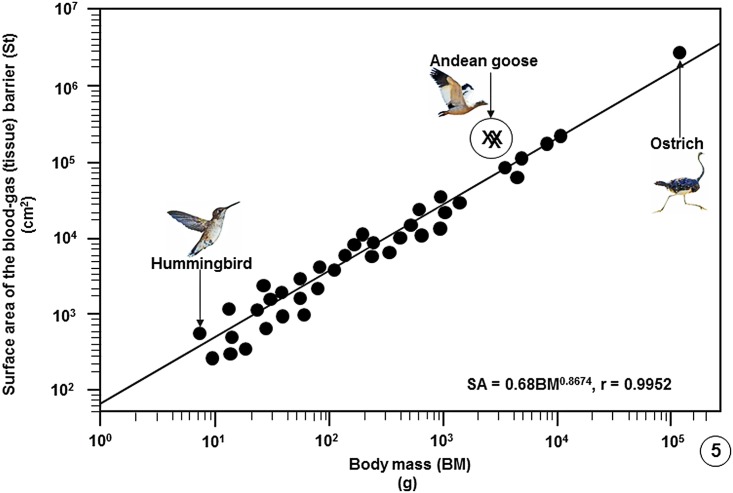
Regression line plotted on logarithmic x-y axes showing the correlation between the surface areas of the blood-gas (tissue) barriers (St) of the lungs of birds on which data are available against Body Masses (BM). The values of the three specimens of the Andean goose (*Chloephaga melanoptera*) that were investigated in this study lie above the regression line of the bird population. The data on which the regression line was plotted are summarized in publications [33], [37], [39] and [54–56] and are given in data supporting this paper (S8 Table).

**Fig 6:**
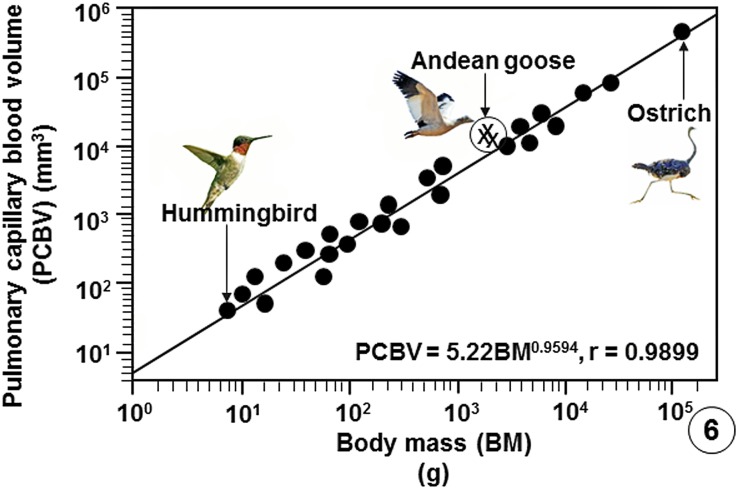
Regression line plotted on logarithmic x-y axes showing the correlation between the Pulmonary Capillary Blood Volume (PCBV) of the lungs of birds that have been studied against Body Masses (BM). The values of the three specimens of the Andean goose (*Chloephaga melanoptera*) that were investigated in this study lie above the regression line of the bird population. The data on which the regression line was plotted are summarized in publications [33], [37], [39] and [54–56] and are given in data supporting this paper (S8 Table).

**Fig 7:**
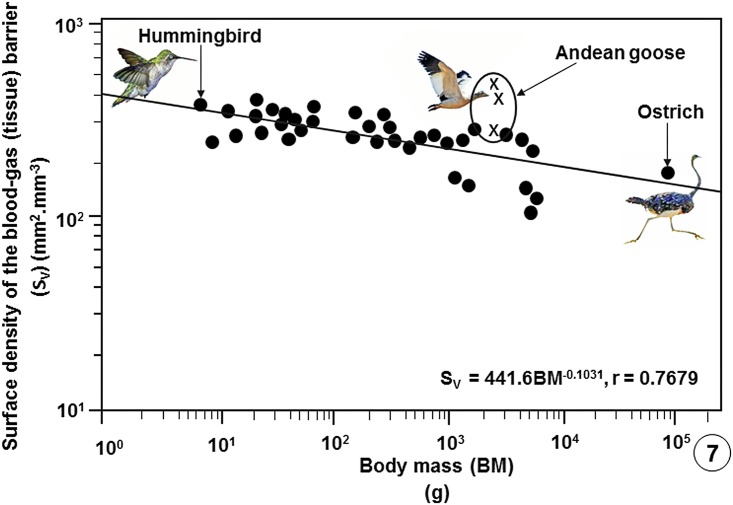
Regression line plotted on logarithmic x-y axes showing the correlation between the surface densities of the blood-gas (tissue) barriers per unit volume of the lung parenchyma (S_V_) of the lungs of the birds that have been studied against Body Masses (BM). The values of the three specimens of the Andean goose (*Chloephaga melanoptera*) investigated in this study lie above the common regression line of the bird population. The data on which the regression line was plotted are summarized in publications [33], [37], [39] and [54–56] and given in data supporting this paper (S8 Table).

**Fig 8:**
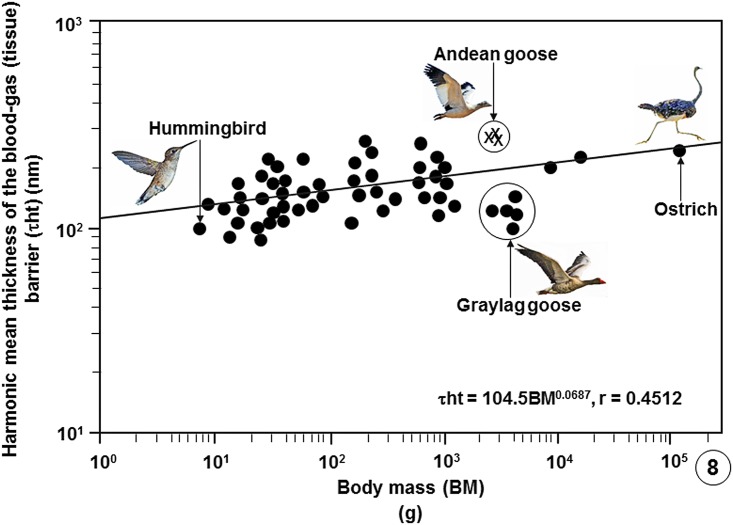
Regression line plotted on logarithmic x-y axes showing the correlation between the harmonic mean thicknesses of the blood-gas (tissue) barriers (τ_ht_) of the lungs of birds that have been studied against Body Masses (BM). The values of the three specimens of the Andean goose (*Chloephaga melanoptera*) investigated in this study lie above the common regression line of the bird population. Showing that the tissue barrier of the Andean goose is not particularly thin, the tissue barrier of the relatively larger, low altitude dwelling greylag goose (*Anser anser*) is relatively much thinner. The data on which the regression line was plotted are summarized in publications [33], [37], [39] and [54–56] and given in data supporting this paper (S8 Table).

**Fig 9:**
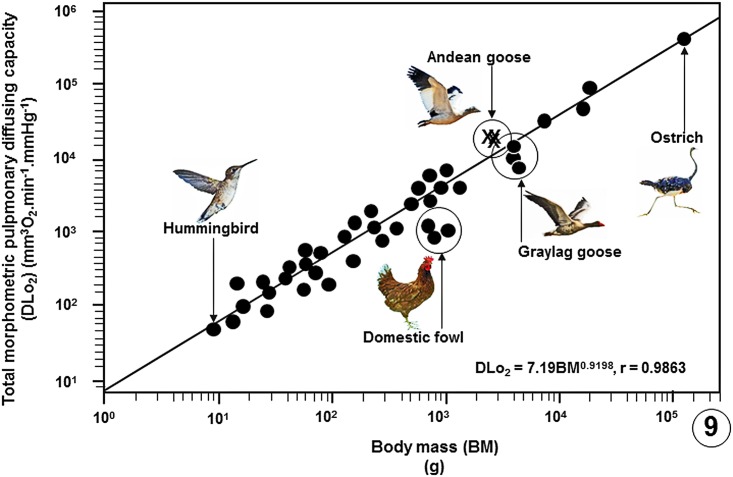
Regression line plotted on logarithmic x-y axes showing the correlation between the total morphometric pulmonary diffusing capacities of the lungs for oxygen (DLo_2_) of the birds that have been studied against Body Masses (BM). The values of the three specimens of the Andean goose (*Chloephaga melanoptera*) that were investigated in this study lie above the common regression line of the bird population. The data on which the regression line was plotted are summarized in publications [33], [37], [39] and [54–56] and given in data supporting this paper (S8 Table).

Comparison of some pulmonary morphometric parameters of the lung of the Andean goose with those of some species of birds is shown on Table 2 (S7 Table) and with those of the population of birds on which data are available (S8 Table) on Figs 4–9.

**Table 2:** Comparison of some pulmonary morphometric parameters of the Andean goose (*Chloephaga melanoptera*) with those of other species of birds. BM, body mass; τ_ht_, harmonic mean thickness of the blood-gas (tissue) barrier; VL.BM^-1^, volume of the lung per unit body mass; V_(PCB)_.BM^-1^, volume of the pulmonary capillary blood per unit body mass; V_(PCB)_.S(t)-1, volume of the pulmonary capillary blood per unit surface area of the blood-gas tissue barrier; S_(t)_.BM^-1^, surface area of the blood-gas (tissue) barrier per unit body mass; S_(t)_.V(LP)-1, surface area of the blood-gas (tissue) barrier per unit volume of the lung parenchyma; DLo_2_.kg^-1^, total (overall) morphometric pulmonary diffusing capacity of the lung per unit body mass.

Common English name/ Latin name	BM (kg)	τ_ht_ (μm)	V(_L)_.BM^-1^ (cm^3^.kg^-1^)	V_(PCB)_.BM^-1^ cm^3^.kg^-1^	V _(PCB)_.S_(t)_^-1^ (cm^3^.m^-2^)	S_(t)_.BM^-1^ (cm^2^.g^-1^)	S_(t)_.V_(LP)_^-1^ (mm^2^.mm^-3^)	DLo_2_.kg^-1^ (mlO_2_.sec^-1^.mbar^-1^.kg^-1^)^Θ^
Andean Goose^a^ (*Chloephaga melanoptera)*	2.64	0.222	42.8	7.44	0.8	96.5	330	0.119
Violet-Eared Hummingbird^b^ (*Colibri coruscans)*	0.007	0.099	42.9	7.00	1.0	87.1	389	-^β^
African Rock Martin^c^ (*Hirundo fuligula)*	0.014	0.090	24.1	5.46	0.7	86.5	353	5.284
House Sparrow^c^ (*Passer domesticus)*	0.026	0.096	29.8	6.31	0.9	63.0	389	3.819
Budgerigar^d^ (*Mellopsitacus undulates)*	0.040	0.117	28.3	4.39	1.1	42.6	317	0.072
Rock Dove^d^ (*Columba livia)*	0.220	0.161	34.3	5.01	1.3	39.8	254	0.122
Spectacled Guillemot^e^ (*Larus argentatus)*	0.740	0.153	27.8	4.34	1.5	22.1	236	0.045
Muscovy Duck^f^ (*Cairina moschata)*	1.630	0.199	30.0	4.31	1.5	30.0	200	0.079
Domestic Fowl^g^ (*Gallus domesticus)*	2.140	0.318	12.6	1.63	1.6	8.7	172	0.020
Graylag Goose^e^ (*Anser anser)*	3.840	0.118	30	3.25	1.4	23.1	253	0.059
Humboldt Penguin^h^ (*Spheniscus humboldti)*	4.500	0.530	30.4	8.02	4.4	18.1	116	0.067
Emu^i^ (*Dromaius novaehollandiae)*	30.00	0.232	36.7	5.55	1.7	5.4	82	0.017
Ostrich^j^ (*Struthio camelus)*	45.00	0.560	38.1	5.46	2.1	30.1	98.3	0.086

Sources of data:

^a^This study;

^b^Dubach [57];

^c^Maina [54];

^d^Maina [33, 36, 37, 39];

^e^Maina [33, 36, 37, 39, 58];

^f^Vidyadaran et al. [52];

^g^Abdalla et al. [49];

^h^Maina and King [61];

^i^Maina and King [55];

^j^Maina and Nathaniel [56].

^**Θ**^: In the lung of the Andean goose on which the revised model [45] was used, the value of the DLo_2_ was 31.6% greater than that determined by the older model [44]. This factor (31.6%) was used to adjust the values for the other species of birds (shown on the table) that had been calculated using the older model [43].

^**β**^: The total morphometric pulmonary diffusing capacity of the violet eared hummingbird was not reported by Dubach [57] nor were sufficient data given to allow the value to be calculated.

## Discussion

This study reports that certain morphological- and morphometric specializations occur in the lung of the Andean goose (*Chloephaga melanoptera*). Such features should increase the gas exchange efficiency of the lung and may explain the high pulmonary O_2_ extraction rates that were recently reported in this species by Lague et al. [28]. The Andean goose is one of the largest birds in the Western Hemisphere that lives at an altitude of above 3000 m in valleys and plateaus of the Andes from Peru to southern Chile. The species is non-migratory and may breed at elevations as high as 5,500 meters but rarely ever descends below 2,000 m [40].

### Morphological findings

Compared with the lungs of the other species of birds that have so far been studied where the infundibulae are avascular [33, 36] (Fig 3A), in the lung of the Andean goose, the structural units are intensely vascularized (Fig 3C–3F). To an undermined extent, this feature should substantially increase the respiratory surface area and the volume of the pulmonary capillary blood (V_(pcb)_). Location of infundibular BCs next to the parabronchial lumen (PL) (Fig 2C–2F) results in the following features that increase respiratory efficiency: (a) the infundibular dead-space air is reduced or possibly eliminated, (b) the infundibular BCs are exposed to inspired air with the highest possible partial pressure of O_2_ and (c) the BCs are not affected by the process of ‘O_2_ screening’ [46, 47].

The significance of the sparse distribution of the interparabronchial septa in the lung of the Andean goose (Figs 1C, 1D and 2A) is unclear. Scheid [48] contended that since no air pressure differences exist between the parabronchi in the avian lung, little if any air moves between them. If the assertion is correct, the sparse distribution of the interparabronchial septa may be of no functional consequence in the lung of the Andean goose. The septa may be a phylogenetic relic carried over during the evolution of the lung. Interparabronchial septa are well-developed in the lungs of the galliform birds [49–53] while they are totally lacking in those of the passerine ones [53, 54].

### Morphometric findings

In the avian lung, the mean volume density of the LP is 49% [33, 36, 39, 52–54]. The values range from 18 to 78% respectively in the lungs of the emu (*Dromaius novaehollandiae*) [55] to that of the ostrich (*Struthio camelus*) [56]. For the Andean goose, the mean value of 62% lies on the upper end of the range in birds. The highest mass-specific respiratory surface areas in birds of 87.1 and 86.5 cm^2^.g^-1^ have respectively been reported in the lungs of a 7.3 g violet-eared hummingbird (*Colibri coruscans*) [57] and a 13.7 g African rock martin (*Hirundo fuligula*) [33, 36, 37, 39, 58]. The value of 96.5 cm^2^.g^-1^ for the lung of the Andean goose is the highest so far reported in a bird (Table 2). The very high mass specific respiratory surface area of 800 cm^2^g^-1^ that was reported for an unnamed species of hummingbird by Stanislaus [59] should be treated with caution since it is not explained how the value was determined.

In vertebrate lungs, respiratory surface area is increased by subdivision of the LP [33, 36, 39] and/or gross enlargement of the lung [60]. Stereologically, surface area (S) per unit volume (V) is designated surface density (S_V_) [43]. The S_V_ of the BGB, i.e., the surface area of the BGB per unit volume of the lung parenchyma (S_(t)_.V_(LP)_^-1^), scales inversely with body mass [33, 36, 39]. S_(t)_.V_(LP)_^-1^ indicates the intensity of the subdivision (compartmentalization) of the LP and therefore the relative sizes (diameters) of the terminal respiratory units in a lung. In the avian lung, the respiratory units (the ACs) range in diameter from 3 μm in the smaller species [54] to 20 μm in that of the ostrich [56]. In this study, while the diameters of the ACs were not directly determined, their sizes corresponded to those of the erythrocytes of ~13 μm (Fig 3C). The large S_(t)_.V_(LP)_^-1^ of 330±70.6 mm^2^.mm^-3^ that exceeds the values of most birds (Table 2) shows that the very small ACs are generated by intense subdivision of the LP. In birds that have so far been investigated, a S_(t)_.V_(LP)_^-1^ of 82 mm^2^.mm^-3^ was reported in the lung of the emu [55] and the highest ones of 389 mm^2^.mm^-3^ in those of the violet-eared hummingbird [57] and the house sparrow (*Passer domesticus*) [33, 36, 39]. The extreme subdivision of the LP of the avian lung explains the unexpected outcome that while the avian lung is 27% smaller compared to that of a mammal of equivalent body mass and the volume density of the LP is about one-half that of a mammalian lung [1, 33, 36, 39], the respiratory surface in a bird lung is ~15% greater.

In birds, the thinnest BGBs (τ_ht_) have been reported in the lungs of the violet-eared hummingbird (0.099 μm) [57], the house sparrow (0.096 μm) and the African rock martin (0.090 μm) [33, 36, 39]. The thickest BGBs occur in the ostrich (0.56 μm) [56] and the Humboldt penguin (*Spheniscus humboldti*) (0.53 μm) [61] lungs. For the Andean goose, the mean τ_ht_ of 0.222 μm was not relatively remarkably thin (Table 2; Fig 8). A τ_ht_ of 0.118 μm was reported in the lung of a 3.84 kg body mass low altitude flying greylag goose (*Anser anser*) [33, 36, 37, 58]. For a bird that lives and flies in a cold and hypoxic environment where heart rate and cardiac output may need to be constantly high, the thicker BGB in the lung of the Andean goose may help avert structural failure of the BGB, a feature that has been reported to occur in the avian lungs [62, 63]. The particularly thick BGB of the lung of the Humboldt penguin [61] and the presence of plentiful connective tissue elements, especially of collagen in the BGB [64], was attributed to the capacity of the lung tolerating high hydrodynamic pressures during dives.

Gas exchangers, including the lungs, are characteristically intensely vascularized [65–69]. The greater the degree of vascularization the larger the volume of blood in the BCs [33, 36, 37, 39, 54]. In the African rock martin, 29% of the volume of the lung consists of blood, with 79% of it located in the BCs [33, 36, 37, 39, 54]. For the Andean goose, the volume of blood comprised 30% of the V_L_, with 65% of it in the BCs. Among the data available on the bird lungs, the mass-specific V_(PCB)_ of the lung of the Andean goose (7.44 cm^3^.kg^-1^) is only surpassed by that of the diving Humboldti penguin (8.02 cm^3^.kg^-1^) [60] (Table 2). The ratio of the V_(PCB)_ to the total respiratory surface (SA), i.e., V_(PCB)_.S_(t)_^-1^, the so-called capillary loading [70], indicates the degree of exposure of pulmonary capillary blood to air [65, 70], with low values indicating high gas exchange efficiency. In the avian lungs that have been investigated, V_(PCB)_.S_(t)_^-1^ ranges from 0.7 cm^3^.m^-2^ in the African rock martin [33, 36, 37, 39, 54] to 4.4 cm^3^.m^-2^ in the Humboldt penguin [55]. For the lung of the Andean goose, the particularly low value of 0.8 cm^3^.m^-2^ points out to a specialization for gas exchange.

Among the data available on the avian lungs, the mass-specific DMo_2_ of the lung of the Andean goose of 1.479 mlO_2_.sec^-1^.mbar^-1^.kg is only surpassed by that of the violet-eared hummingbird [57] while the mass-specific DLo_2_ (0.119 mlO_2_.sec^-1^.mbar^-1^.kg) is only surpassed by that of the lung of the African rock martin [33, 36, 37, 39, 54] (Table 2). Because the DLo_2_ integrates the diffusing capacities, i.e., the conductances, of the structural components that form the air-hemoglobin pathway [42–45], structurally, the parameter it is the most comprehensive estimator of the gas exchange capacity of a lung. The large mass-specific DLo_2_ of the lung of the Andean goose is consistent with and undoubtedly contributes to the high pulmonary O_2_ extraction rates reported for this species by Lague et al. [28].

Compared with data on lungs of species of bird that have so far been investigated, the Andean goose has relatively larger lungs (Fig 4), larger surface area of the BGB (Fig 5), larger V_(PCB)_ (Fig 6), greater surface density of the BGB (Fig 7) and the BGB is moderately thin (Fig 8). These specializations have generated relatively larger DLo_2_ of the lung of the Andean goose (Fig 9).

#### Comment on the modeling of the oxygen diffusing capacity of the avian lung and future research directions

Here, the revised model of Weibel et al. [45] was used to determine the DMo_2_ and the DLo_2_ of the lung of the Andean goose. To compare the value of the DLo_2_ of the lung of the Andean goose with those of species of birds that had been calculated using the older model of Weibel [44], the values were adjusted by a factor of 31.6%. This was the ratio of the value of the DLo_2_ of the Andean goose calculated by the revised model of Weibel et al. [45] to that calculated by the older model of Weibel [44]. Because the current study is the only one where the two models have been applied and compared on the lung of a species of bird, the adjustment factor should be considered tentative. Taking into account the large structural and functional differences between the avian (parabronchial) lung and the mammalian (bronchioalveolar) one, it was considered judicious to adjust the avian DLo_2_ values that had been determined with the older model of Weibel [44] with an avian lung derived adjustment factor rather than by applying the adjustment factors that were derived from the mammalian lung in Weibel et al. [45]. The application of the two models on the avian lung should be assessed on lungs of a larger number of species birds.

Following this study, it will be of great interest to find out whether the pulmonary specializations that have been found in the lung of the Andean goose characterize the lungs of all high altitude flying birds or different adaptive strategies exist in the lungs of the other birds that fly to high elevations.

## Supporting information

S1 Table Absolute volumes of the main structural components of the lungs of the three specimens of the Andean goose, *Chloephaga melanoptera*. (DOCX)

S2 Table Volume densities of the main structural components of the lung of the Andean Goose, *Chloephaga melanoptera*. (DOCX)

S3 Table Volume densities (V_V_) and absolute volumes (V) of the components of the exchange tissue: the Air Capillaries (AC), the Blood Capillaries (BC); the Structural Tissue (ST) of the parenchyma of the lungs of the Andean goose and the Pulmonary Capillary Hematocrit (PCH). (DOCX)

S4 Table Surface areas of the Blood-Gas Barrier (BGB), the total surface area of the air- and the blood capillaries (AC+BC), Red Blood Cells (RBC) and the Capillary Endothelium (CE). (DOCX)

S5 Table Harmonic mean thicknesses (μm) of the blood-gas (tissue) barrier (τht) and the harmonic mean thickness of the total barrier, i.e., the distance between the respiratory surface and the erythrocyte membrane (τhb), the air-hemoglobin pathway. (DOCX)

S6 Table Mean pulmonary diffusing capacities, namely the diffusing capacity of the blood-gas tissue barrier (Dto_2_), the membrane (Dmo_2_), the erythrocytes (Deo_2_) and the total morphometric diffusing capacity (DLo_2_). (DOCX)

S7 Table Surface area of the blood-gas barrier per unit body mass, surface area of the blood-gas barrier per unit volume of the exchange tissue, pulmonary capillary blood volume per unit surface area of the blood-gas barrier, volume of the lung per unit body mass, diffusing capacity of the blood-gas barrier per unit body mass and total pulmonary diffusing capacity per unit body mass. (DOCX)

S8 Table Pulmonary morphometric parameters of the bird lung on which the graphs (regression lines) were plotted and the sources of data. (DOCX)
