# Supplementary material for: Morphological and morphometric specializations of the lung of the Andean goose, Chloephaga melanoptera: A lifelong high-altitude resident
Source: PLoS One. 2017 Mar 24;12(3):e0174395. doi: 10.1371/journal.pone.0174395 (PMC5365123; doi:10.1371/journal.pone.0174395)
Supplement: S1 Table — (DOCX) [file pone.0174395.s001.docx]

| Specimen number | Body mass  (g) | Volume of the lung  (cm^3^) | Volume of the exchange tissue  (cm^3^) | Volume of the lumina of the secondary bronchi and parabronchi (cm^3^) | Volume of the blood vessels larger than capillaries  (cm^3^) | Volume of the primary bronchus  (cm^3^) |
| --- | --- | --- | --- | --- | --- | --- |
| 1 | 2800 | 109.02 | 67.27 | 28.13 | 10.37 | 3.28 |
| 2 | 2530 | 118.41 | 73.06 | 30.55 | 11.26 | 3.56 |
| 3 | 2580 | 109.72 | 67.70 | 28.31 | 10.43 | 3.30 |
| Mean±SD | 2636.67±144 | 112±5.23 | 69.30±3.23 | 29.0±1.35 | 10.7±0.50 | 3.38±0.16 |

**S1 Table:** Absolute volumes^#^ of the main structural components of the lungs of the three specimens of the Andean goose, *Chloephaga melanoptera*.

^#^ Calculated by multiplying the volume densities of the structural components with the volumes of the lung.
