# Supplementary material for: Morphological and morphometric specializations of the lung of the Andean goose, Chloephaga melanoptera: A lifelong high-altitude resident
Source: PLoS One. 2017 Mar 24;12(3):e0174395. doi: 10.1371/journal.pone.0174395 (PMC5365123; doi:10.1371/journal.pone.0174395)
Supplement: S2 Table — (DOCX) [file pone.0174395.s002.docx]

**S1 Table:** Volume densities (%) of the main structural components of the lung of the Andean Goose, *Chloephaga melanoptera*

| Region | Exchange tissue  (Lung parenchyma) | Lumina of the secondary bronchi and parabronchus | Blood vessels larger than capillaries | Primary bronchus |
| --- | --- | --- | --- | --- |
| R1 (1) | 65.20 | 24.30 | 10.50 | 0.00 |
| R2 (1)  (2) | 62.40  64.90 | 29.10  24.0 | 8.50  11.10 | 0.00  0.00 |
| R3 (1)  (2) | 48.54  69.31 | 26.3  22.0 | 9.41  8.69 | 15.75  0.00 |
| R4 (1)  (2) | 59.80  68.50 | 24.80  22.20 | 12.60  9.38 | 2.81  0.00 |
| R5 (1)  (2) | 55.20  65.80 | 30.80  24.30 | 8.19  9.90 | 5.81  0.00 |
| R6 (1)  (2) | 50.10  68.70 | 33.40  22.80 | 7.81  8.50 | 8.69  0.00 |
| Mean±SD | 61.70±7.39 | 25.80±3.73 | 9.51±1.43 | 3.01±5.16 |

R - Regions of the slices of the lung of one of the specimens
