# Supplementary material for: Morphological and morphometric specializations of the lung of the Andean goose, Chloephaga melanoptera: A lifelong high-altitude resident
Source: PLoS One. 2017 Mar 24;12(3):e0174395. doi: 10.1371/journal.pone.0174395 (PMC5365123; doi:10.1371/journal.pone.0174395)
Supplement: S3 Table — (DOCX) [file pone.0174395.s003.docx]

**S3 Table:** Volume densities (V_V_) and absolute volumes (V) of the components of the exchange tissue: the air capillaries (AC), the blood capillaries (BC); the structural tissue (ST) of the parenchyma of the lungs of the Andean goose and the pulmonary capillary hematocrit (PCH).

| Specimen | Air capillaries | | Blood capillaries | | Structural tissue | | Pulmonary capillary hematocrit^*^ |
| --- | --- | --- | --- | --- | --- | --- | --- |
|  | V_Vac_  (%) | V_ac_  (cm^3^) | V_VBC_  (%) | V_BC_  (cm^3^) | V_VST_  (%) | V_ST_  (cm^3^) | PCH (%) |
| 1 | 65.67 | 44.18 | 26.00 | 17.49 | 8.33 | 5.60 | 63.3 |
| 2 | 64.50 | 47.12 | 27.17 | 19.85 | 8.33 | 6.09 | 65.3 |
| 3 | 60.03 | 40.64 | 31.41 | 21.26 | 8.56 | 5.80 | 69.6 |
| Mean±SD | 63.41±3.0 | 43.98±3.3 | 28.19±2.9 | 19.50±1.9 | 8.41±0.13 | 5.83±2.5 | 66.07±3.22 |

^*^The pulmonary capillary hematocrit is the volume density of the erythrocytes in the blood capillaries
