# Supplementary material for: Morphological and morphometric specializations of the lung of the Andean goose, Chloephaga melanoptera: A lifelong high-altitude resident
Source: PLoS One. 2017 Mar 24;12(3):e0174395. doi: 10.1371/journal.pone.0174395 (PMC5365123; doi:10.1371/journal.pone.0174395)
Supplement: S4 Table — (DOCX) [file pone.0174395.s004.docx]

**S4 Table:** Surface areas of the blood-gas barrier (BGB), the total surface area of the air- and the blood capillaries (AC+BC), red blood cells (RBC) and the capillary endothelium (CE).

| Specimen | BGB | AC+BC | RBC | CE |
| --- | --- | --- | --- | --- |
| 1 | 223,467.65 | 299,597.60 | 217,561.02 | 224,616.17 |
| 2 | 270,143.80 | 356,390.24 | 274,598.68 | 284,755.80 |
| 3 | 263,113.19 | 316,941.67 | 319,235.21 | 289,422.45 |
| Mean ±SD | 252,241.51±25,200 | 324,309.83±29,100 | 270,464.97±5,100 | 266,264.80±3,610 |
