# Supplementary material for: Morphological and morphometric specializations of the lung of the Andean goose, Chloephaga melanoptera: A lifelong high-altitude resident
Source: PLoS One. 2017 Mar 24;12(3):e0174395. doi: 10.1371/journal.pone.0174395 (PMC5365123; doi:10.1371/journal.pone.0174395)
Supplement: S5 Table — (DOCX) [file pone.0174395.s005.docx]

**S5 Table:** Harmonic mean thicknesses (µm) of the blood-gas (tissue) barrier (τht) and the harmonic mean thickness of the total barrier, i.e., the distance between the respiratory surface and the erythrocyte membrane (τhb), the air-hemoglobin pathway.

| Specimens | τht | τhb |
| --- | --- | --- |
| 1 | 0.222 | 0.427 |
| 2 | 0.210 | 0.429 |
| 3 | 0.234 | 0.565 |
| Mean ±SD | 0.222±0.012 | 0.474±0.080 |
