# Supplementary material for: Morphological and morphometric specializations of the lung of the Andean goose, Chloephaga melanoptera: A lifelong high-altitude resident
Source: PLoS One. 2017 Mar 24;12(3):e0174395. doi: 10.1371/journal.pone.0174395 (PMC5365123; doi:10.1371/journal.pone.0174395)
Supplement: S6 Table — (DOCX) [file pone.0174395.s006.docx]

**S6 Table:** Mean pulmonary diffusing capacities, namely the diffusing capacity of the blood-gas tissue barrier (Dto_2_), the membrane (Dmo_2_), the erythrocytes (Deo_2_) and the total morphometric diffusing capacity (DLo_2_).

| Specimen | Deo_2_ | Dmo_2_ | DLo_2_ |
| --- | --- | --- | --- |
| 1 | 0.198 | 3.28 | 0.280 |
| 2 | 0.224 | 4.89 | 0.325 |
| 3 | 0.240 | 3.44 | 0.330 |
| Mean ±SD | 0.221±0.021 | 3.87±0.887 | 0.312±0.028 |
