# Supplementary material for: Morphological and morphometric specializations of the lung of the Andean goose, Chloephaga melanoptera: A lifelong high-altitude resident
Source: PLoS One. 2017 Mar 24;12(3):e0174395. doi: 10.1371/journal.pone.0174395 (PMC5365123; doi:10.1371/journal.pone.0174395)
Supplement: S7 Table — (DOCX) [file pone.0174395.s007.docx]

| Specimen | S_BGB_.BM^-1^ | S_(A)_.V_(LP)_^-1^ | V_(PCB)_.S_(A)_^-1^ | VL.BM^-1^ | Vc.BM^-1^ | DLo_2_.BM^-1^ |
| --- | --- | --- | --- | --- | --- | --- |
|  | (cm^2^.g) | (mm^2^.mm^3^) | cm^3^.m^-2^ | cm^3^.kg^-1^ | cm^3^.kg^-1^ | mlO_2_.sec^-1^. mbar^-1.^kg-1 |
| 1 | 79.81 | 347.06 | 0.783 | 38.94 | 6.246 | 0.100 |
| 2 | 106.78 | 252.99 | 0.735 | 46.80 | 7.846 | 0.129 |
| 3 | 102.76 | 391.16 | 0.802 | 42.53 | 8.240 | 0.128 |
| Mean ±SD | 96.50±14.6 | 330.40±70.6 | 0.773±0.04 | 42.8±3.9 | 7.444±1.056 | 0.119±0.02 |

**S7 Table:** Surface area of the blood-gas barrier per unit body mass (S_BGB_.BM^-1^), surface area of the blood-gas barrier per unit volume of the exchange tissue (S_(A)_.V_(LP)_^-1^), pulmonary capillary blood volume per unit surface area of the blood-gas barrier (V_(PCB)_.S_(A)_^-1^), volume of the lung per unit body mass (VL.BM^-1^), diffusing capacity of the blood-gas barrier per unit body mass (Dto_2_.BM^-1^) and total pulmonary diffusing capacity per unit body mass (DLo_2_.BM^-1^).
