# Supplementary material for: Morphological and morphometric specializations of the lung of the Andean goose, Chloephaga melanoptera: A lifelong high-altitude resident
Source: PLoS One. 2017 Mar 24;12(3):e0174395. doi: 10.1371/journal.pone.0174395 (PMC5365123; doi:10.1371/journal.pone.0174395)
Supplement: S8 Table — (DOCX) [file pone.0174395.s008.docx]

**S8 Table:** Pulmonary morphometric parameters of the bird lung on which the graphs (regression lines) were plotted and the sources of data

| Order, Scientific andCommon Name | Numbers  n | Body mass  (BM) | Surface area of the blood-gas (tissue) barrier  (SA) | Harmonic mean thickness of the blood-gas (tissue) barrier  (τ_ht_) | Lung volume  (VL) | Volume of the pulmonary capillary blood  (Vc) | Total morphometric pulmonary diffusing capacity  (DLo_2_) |
| --- | --- | --- | --- | --- | --- | --- | --- |
| Units |  | (kg) | (m^2^) | (µm) | cm^3^ | cm^3^ | mlO_2_.sec^-1^.mbar^-1^ |
| **STRUTHIOFORMES**Struthio camelus Ostrich^a^ | 1 | 45 | 120.34 | 0.560 | 1,563 | 250 | 2.93 |
| **SPHENISCIFORMES**Spheniscus humboldti Humboldt penguin^b^ | 1 | 4.5 | 8.20 | 0.530 | 1,368 | 358 | 0.231 |
| ANSERIFORMES Anas platyrhynchos Mallard duck^c^ Anser anserGreylag goose^d^ | 5  5 | 1.04  3.84 | 2.97  8.87 | 0.113  0.118 | 30.60  95.30 | 4.1  12.5 | 0.050  0.172 |
| FALCONIFORMES Falco tinnunculus Common kestrel^e^ | 1 | 0.07 | 0.53 | 0.210 | 3.10 | 0.411 | 0.005 |
| GALLIFORMES  *Gallus gallus*  Domestic fowl^f^ | 3 | 2.14 | 2.16 | 0.318 | 27.00 | 3.50 | 0.032 |
| CHARADRIIFORMES Alca tordaRazorbill^g^Cephus carbo Spectacled guillemot^h^ Larus argentatusHerring gull^i^Larus canus Common gull^j^ Larus ridibundus Black-headed gull^k^ | 2  9  2  1  6 | 0.49  0.74  0.65  0.30  0.25 | 2.40  1.93  1.46  0.63  0.61 | 0.230  0.193  0.153  0.116  0.146 | 18.10  24.10  18.20  7.10  7.50 | 2.20  3.20  2.13  0.67  0.78 | 0.029  0.043  0.025  0.010  0.011 |
| COLUMBIFORMES Columba livia Rock dove^l^ | 1 | 0.22 | 0.86 | 0.161 | 7.40 | 1.10 | 0.020 |
| Streptopelia decaocta Collared turtle dove^m^ Streptopelia senegalensis Laughing dove^n^ | 16  1 | 0.19  0.06 | 0.84  0.27 | 0.218  0.227 | 6.50  1.80 | 0.78  0.33 | 0.012  0.005 |
| PSITTACIFORMES Melopsittacus undulatus Budgerigar^o^ | 6 | 0.036 | 0.15 | 0.117 | 1.03 | 0.16 | 0.002 |
| CUCULIFORMES Chrysococcyx klaas Klaa’s cuckoo^p^ | 3 | 0.027 | 0.09 | 0.157 | 0.66 | 0.12 | 0.002 |
| COLIIFORMES Colius striatus Spectacled mousebird^q^ | 1 | 0.051 | 0.10 | 0.148 | 0.71 | 0.132 | 0.002 |
| PICIFORMES Pogoniulus bilineatus Golden-rumped tinkerbird^r^ | 2 | 0.015 | 0.03 | 0.165 | 0.23 | 0.050 | 0.001 |
| CASUARIIFORMES  *Dromaius novaehollandiae*  Emu^s^ | 1 | 0.030 | 6.28 | 0.232 | 1,100 | 27.74 | 0.005 |
| PASSERIFORMES Ambryospiza albifrons Grosbeak weaver^t^ Cisticola cantans Singing cisticola^t^ Hirundo fuligula African rock martin^t^ Lanius collaris Fiscal shrike^t^ Passer domesticus House sparrow^t^ Ploceus baglafecht Baglafecht weaver^t^ Prinia subflava Twany prinia^t^ Sturnus vulgarisCommon starling^t^Turdus iliacusRedwing^t^Turdus olivaceus Olive thrush^t^ | 5  4  1  6  12  6  1  10  1    2 | 0.037  0.015  0.014  0.033  0.026  0.033  0.091  0.073  0.051  0.065 | 0.14  0.04  0.12  0.12  0.17  0.12  0.03  0.36  0.17  0.24 | 0.121  0.122  0.090  0.170  0.096  0.151  0.124  0.141  0.120  0.127 | 0.97  0.31  0.33  0.72  0.76  0.88  0.21  2.02  1.19  1.40 | 0.200  0.060  0.070  0.140  0.160  0.160  0.040  0.270  0.213  0.310 | 0.003  0.001  0.003  0.002  0.003  0.002  0.001  0.005  0.003  0.005 |

**Sources:**

| a | Maina JN, Nathaniel C. A qualitative and quantitative study of the lung of an ostrich, *Struthio camelus*. J  Exp Biol. 2001; 204: 2313-2330. |
| --- | --- |
| b | Maina JN, King AS. A morphometric study of the lung of a Humboldt penguin *(Spheniscus humboldti*). Zentral Vet Med Ser C, Anat Histol Embryol. 1987; 16: 293-297. |
| c | Maina JN. The morphometry of the avian lung. In: King AS, McLelland J, editors. Form and function in birds, Vol.4. London: Academic Press; 1989. pp. 307-368.  Maina JN. The design of the lung-air sac system of birds: development, structure, and function. Heidelberg: Springer-Verlag; 2005.  Maina JN, King AS, Settle G. An allometric study of the pulmonary morphometric parameters in birds,  with mammalian comparison. Phil Trans R Soc Lond. 1989; 326B: 1-57. |
